# Becoming More Precise in Psychiatry: Signposts, Destinations, Maps and Territories

**DOI:** 10.1002/hup.70013

**Published:** 2025-07-23

**Authors:** Milica M. Nestorovic, David S. Baldwin

**Affiliations:** ^1^ University Clinical Center of Serbia Clinic of Psychiatry Belgrade Serbia; ^2^ Faculty of Medicine Clinical and Experimental Sciences University of Southampton Southampton UK

The metaphorical expression ‘*Don't mistake the signpost for the destination*’ cautions against the dual pitfalls of becoming over‐preoccupied with the details of a route and of being prematurely encouraged by interim way‐posts, when undertaking an adventurous journey towards a cherished goal. In psychiatric practice, patients with mental health problems and their attending professionals from varying disciplines still yearn for the targeted discovery and routine implementation of individually tailored approaches, designed to shorten the course of illness and improve other clinical outcomes. This personalised medicine approach, which underpins the ‘precision psychiatry’ movement (Fernandes et al. 2017), has the laudable ambition of ‘getting the right treatment to the right patient at the right time’, and has gained additional momentum through the recent publication of the Precision Psychiatry Roadmap (PPR) (Kas et al. 2025), constructed by a consortium including patient organisations, clinicians, scientists, industrial representatives and regulatory agencies. Its carefully crafted eight sub‐components, five phases (envisaged to occur over at least 15 years) and 10 anticipated future implementation steps together provide a detailed plan, for incorporating biology‐informed evidence into symptom‐based diagnoses so allowing for the discovery, development and deployment of mechanism‐based treatments for patients with mental disorders, designed to be more effective and acceptable than current approaches. So, how useful might such a ‘map’ be?

It is worth considering two fundamental challenges. First, idioms of psychological distress vary between individuals and across cultures, patients offer psychological complaints to clinicians in variable ways, and health professionals differ in their preferred style in asking direct questions about key symptoms necessary for accurate psychiatric diagnosis. For example, even within a single country, there is worrying variation between mental health services in the comprehensiveness of patient assessment in anxiety and depressive disorders, leading to concerns about the validity and reliability of recorded psychiatric diagnosis (Baldwin et al. 2021). In addition, reporting of psychological symptoms is affected by illness‐related difficulties in recollection and processing of emotional information (Jongs et al. 2022). It seems probable that ‘biology‐informed evidence’ could only help to supplement and refine treatment decisions based on symptom‐based diagnoses when symptoms are sought and evaluated in a standardised but culturally sensitive manner, through structured clinical interviews and robust psychometric rating scales. Second, whilst the PPR authors rightly acknowledge the importance of evaluating the individual ‘exposome’—which incorporates detailed consideration of personal factors such as socioeconomic status, lived environment, trauma exposure, ‘lifestyle’, inflammation and gut microbiome—experience from other areas of medicine including oncology suggests that the development of reliable instruments for evaluating such variable and dynamic entities which evolve over the course of a lifetime is ‘extremely challenging’ (Wild 2005).

In addition, the PPR authors emphasise the pivotal importance of integrating biomarkers into psychiatric practice, in underpinning the future success of the roadmap approach to precision psychiatry: ‘*The goal of precision psychiatry cannot be achieved without biomarkers*’ (Kas et al. 2025). A ‘biomarker’ can be defined as a characteristic that is measured as an indicator of normal biological processes, pathogenic processes, or biological responses to an exposure or intervention; in psychotropic drug development, biomarkers might underpin diagnosis, monitoring, prognosis, treatment identification and tolerability and safety considerations. Biomarker identification and evaluation has advanced diagnosis, staging and drug development in Alzheimer's disease, thereby contributing to approval by the United States Food and Drug Administration of a monoclonal antibody targeting amyloid plaques as a treatment for the condition (Harris 2023). It is to be hoped that similar advances could facilitate development and regulatory approval of compounds for other neuropsychiatric conditions. The chances of this success rest not only on analytical validation (i.e. the marker accurately measures what it purports to measure, with necessary sensitivity and specificity) and clinical validation (i.e. the marker reflects underlying processes of interest and has predictive validity in independent samples), both emphasised by the PPR authors, but also on other key factors such as acceptability to patients, ease of incorporation in routine clinical practice, and cost‐effectiveness in determining clinical decisions. An identified biomarker would only be adopted widely if it could be used easily.

The above reservations might seem a little churlish: without doubt, the PPR is a most valuable initiative. Its dispassionate but persuasive call for a global alignment on principles and procedures, for building consensus on predictive validity from emerging data, and for operationalizing new knowledge into an evolving framework provides much‐needed clarity on steps to take forward into what had previously seemed to be a murky area. The authors emphasise the importance of harmonising research assessments and approaches across different diagnostic populations, recognising that a key first step is active engagement and two‐way communication with multiple stakeholders, whilst acknowledging potential barriers such as the marked disparity in infrastructure between countries and in the variables collected across different study sites.

The Polish‐American scientist and philosopher Alfred Korzybski is credited with another metaphorical expression, ‘*The map is not the territory*’, designed to recognise the difference between the actual world and the world as we understand it: a map of the world is not the same as the world itself. A roadmap can show a landscape, with all its highways and obstacles, peaks and troughs, but is not itself the promised land: however, without access to an instructive directional roadmap, the traveller is likely to either remain static or at risk of becoming lost. The Precision Psychiatry Roadmap (Kas et al. 2025) therefore represents an important, concerted, collective endeavour, to be undertaken over many years, which should be supported by all those who have an interest in advancing understanding of the neuropsychobiology of mental health problems and in the development of novel treatments leading to improved clinical outcomes.

## Conflicts of Interest

David S. Baldwin is Editor‐in‐Chief of *Human Psychopharmacology*. Milica M. Nestorovic declares no conflicts of interest.

## Data Availability

The authors have nothing to report.
